# Vulvovaginal Candidiasis: A Current Understanding and Burning Questions

**DOI:** 10.3390/jof6010027

**Published:** 2020-02-25

**Authors:** Hubertine M. E. Willems, Salman S. Ahmed, Junyan Liu, Zhenbo Xu, Brian M. Peters

**Affiliations:** 1 Department of Clinical Pharmacy and Translational Science, College of Pharmacy, University of Tennessee Health Science Center, Memphis, TN 38163, USA; hwillems@uthsc.edu (H.M.E.W.); jliu81@uthsc.edu (J.L.); zxu43@uthsc.edu (Z.X.); 2 School of Food Science and Engineering, South China University of Technology, Guangzhou 510641, China; sahmed22@uthsc.edu

**Keywords:** vaginitis, vulvovaginal, *Candida*, vagina, VVC, fungal, candidiasis

## Abstract

*Candida albicans*, along with other closely related *Candida* species, are the primary causative agents of vulvovaginal candidiasis (VVC)—a multifactorial infectious disease of the lower female reproductive tract resulting in pathologic inflammation. Unlike other forms of candidiasis, VVC is a disease of immunocompetent and otherwise healthy women, most predominant during their child-bearing years. While VVC is non-lethal, its high global incidence and profound negative impact on quality-of-life necessitates further understanding of the host and fungal factors that drive disease pathogenesis. In this review, we cover the current state of our understanding of the epidemiology, host response, fungal pathogenicity mechanisms, impact of the microbiome, and novel approaches to treatment of this most prevalent human candidal infection. We also offer insight into the latest advancements in the VVC field and identify important questions that still remain.

## 1. Pathology and Epidemiology of Vulvovaginal Candidiasis

Vulvovaginal candidiasis (VVC), is an exceedingly common mucosal infection of the lower female reproductive tract (FRT), caused mostly by the polymorphic opportunistic fungus *Candida albicans*. A member of the normal human microbiota, *C. albicans* commonly colonizes the vaginal lumen asymptomatically [1]. However, symptomatic infection can result from exuberant mucosal inflammation that is caused primarily by fungal overgrowth in the vagina and subsequent epithelial invasion and production of virulence effectors. Common disease symptoms include vaginal itching, burning, pain and redness. Often, these are accompanied by a vaginal discharge consisting of sloughed epithelium, immune cells, yeast, and vaginal fluid. VVC is the most prevalent human candidal infection, estimated to afflict approximately 75% of all women at least once in their lifetime [2]. Moreover, recurrent VVC (RVVC, defined as >3 episodes per year) affects nearly 8% of women globally [3]. Frequently, RVVC requires antifungal maintenance therapy with azole drugs to attenuate disease reemergence [1]. Static activity of the azoles and inadequate immune-mediated clearance are key drivers of disease recurrence. Risk factors for VVC are the use of antibiotics, sexual activity, high-estrogen containing oral contraceptives, pregnancy, use of sodium glucose cotransporter 2 (SGLT2) inhibitors, and uncontrolled diabetes mellitus [4,5]. Risk factors for RVVC are currently unknown, although genome-wide association studies have begun to unravel some genetic determinants of susceptibility (as discussed in depth below). 

In contrast to invasive and oral candidiasis, R/VVC is a disease of immunocompetent and otherwise healthy women [6]. Thus, the global disease burden is much higher for VVC than these other infectious routes. Using rough estimates of susceptible global populations and incidence rates for each of these disease states, invasive candidiasis causes ~700,000 cases per year, oral candidiasis results in ~15.5 million infections per year, and RVVC alone causes approximately 140M cases per year (Table 1). The incidence rate for acute VVC is practically impossible to estimate, given that it is underreported to clinicians due to largely effective over-the-counter treatment options [7]. While VVC is non-lethal, the sheer enormity of disease burden results in ~$1.8B in medical costs each year and the economic impact due to lost work hours was recently extrapolated to approach an additional $1B per annum in the US alone [3,7].

While *C. albicans* is the causative agent of over 90% of VVC cases, other non-*albicans Candida* (NAC) species have been identified as etiological agents. In some instances, the prevalence of NAC species is disproportionately high, exceeding 50% [18]. Of the NAC species, *C. glabrata* is regarded as the second leading cause of VVC (~8% of cases), while *C. krusei*, *C.parapsilosis*, and *C. tropicalis* make up a majority of the remainder [18,19,20]. Vaginal symptoms resulting from infection with NAC species are often reported as being milder than those experienced during VVC caused by *C. albicans* [21]. However, inherent resistance to the azole drug class, as well as acquired resistance mechanisms, can complicate treatment of the NAC species [22,23]. Often, prolonged antifungal regimens or alternative treatment approaches (e.g., vaginal boric acid suppositories) are required for clearance [24,25]. Given reduced therapeutic efficacy, recent reports suggesting increased incidence rates of NAC-associated VVC are somewhat unsettling [26,27]. However, such reports should be taken with caution, as symptomatic VVC can mimic several disease states of the lower FRT and the ability to distinguish *Candida* as a true pathogen from an asymptomatic colonizer can be difficult. Many such reports identifying high proportions of NAC species come from tertiary vaginitis clinics, focused on patients who have failed conventional antifungal therapy [24,28]. Thus, these studies may overestimate the prevalence of NAC species in the general population. One explanation for increased NAC species causing VVC is the use of over-the-counter azole creams for VVC which could lead to the selection of these inherently antifungal resistant species, supporting the above observation [4,29]. 

Prior and recent work using an animal model of infection demonstrated that representative NAC species isolates are incapable of driving vaginal inflammation similar to that of *C. albicans* [30,31]. This was attributed to a lack of robust virulence mechanisms (as described in more detail below). However, it is somewhat difficult to reconcile these results with the relatively large incidence of *C. glabrata* vaginitis [18]. Thus, it is possible that significant strain-to-strain heterogeneity of virulence or metabolism exists amongst clinical isolates or differential mucosal sensitivity to fungi that could help explain NAC-associated VVC. Because the NAC species are comparatively understudied, the remainder of this review will focus largely on disease pathogenesis and therapy through the lens of *C. albicans*.

Lastly, the influences of host physiological processes on the development of VVC remain unclear. However, estrogen clearly plays a major role in governing disease susceptibility [32]. All animal models of VVC (mouse, rat, pig, macaque) require administration of exogenous estrogen to maintain the vaginal fungal burden, inducing keratinization and cornification of the upper squamous epithelial layer [33]. Estrogen susceptibility to VVC is also reflected clinically. Women in their child-bearing years and estrogenic-phases of their reproductive cycle are most susceptible to disease onset. However, prepubescent girls and post-menopausal women are infrequently afflicted, unless they undergo hormone replacement therapy [33]. Whether this susceptibility is exerted through effects on host immune function, vaginal epithelium stratification, or the fungus itself, are still unknown but all have been reported to potentially play a role [34,35]. 

## 2. Fungal Pathogenicity Mechanisms and Host Response

### 2.1. VVC as an Immunopathology

For decades, VVC was regarded by many as the result of a defective adaptive immune response. However, several cross-sectional clinical studies revealed that HIV+ women (with reduced CD4+ T-cell counts) were at no greater risk for developing VVC as compared to healthy controls [6,36,37,38]. This, coupled with animal model data demonstrating no robust role for cell-mediated or humoral immune responses, clearly pointed toward an alternative explanatory mechanism [39]. A groundbreaking study using human volunteers intravaginally challenged with live *C. albicans* revealed that VVC was actually mediated via innate immune responses, in which neutrophil recruitment into the vaginal lumen was positively correlated with reported disease symptoms [40]. Due to the negative impact of host immunity on disease progression, VVC was labeled as an immunopathology. Recognition of the importance of the innate response in driving inflammatory responses associated with VVC has led to many recent insights regarding disease pathogenesis.

As described above, *C. albicans* is polymorphic, adopting two major morphological forms: the ovoid yeast and elongated hypha [41]. It has long been observed that the capacity to transition between these morphologies is the primary virulence attribute of *C. albicans*, as strains unable to undergo this switch are severely attenuated in pathogenicity or colonization [42]. Surprisingly, the use of hypha-deficient transcriptional regulator mutants (e.g., Δ/Δ*efg1* and Δ/Δ*efg1*/*cph1*) in a murine model of VVC revealed elevated colonization as compared to hypha-competent strains [43]. However, the capacity to elicit neutrophil recruitment, mucosal damage (measured by LDH release), innate inflammatory cytokine (e.g., Interleukin-1β), or alarmins (e.g., S100A8) was significantly reduced with these mutants. These results raised two questions: “Do neutrophils help clear the fungus?” and “Are recruited neutrophils themselves causative agents of host damage (as is observed for other common immunopathologies such as arthritis and asthma)?” However, depletion of neutrophils with neutralizing antibodies neither reduced fungal load nor vaginal LDH levels during infection, suggesting that neutrophils are non-protective under such conditions and that mucosal damage is mediated by the fungus [43]. These results were mirrored in a prior study demonstrating that neutrophil depletion indeed reduced vaginal inflammation during murine VVC, but ultimately did not impact colonization and the course of infection [44]. Clearly, *C. albicans* is capable of eliciting vaginal inflammation, but how?

### 2.2. Sensing Fungi at the Vaginal Mucosa

**Figure 1:**
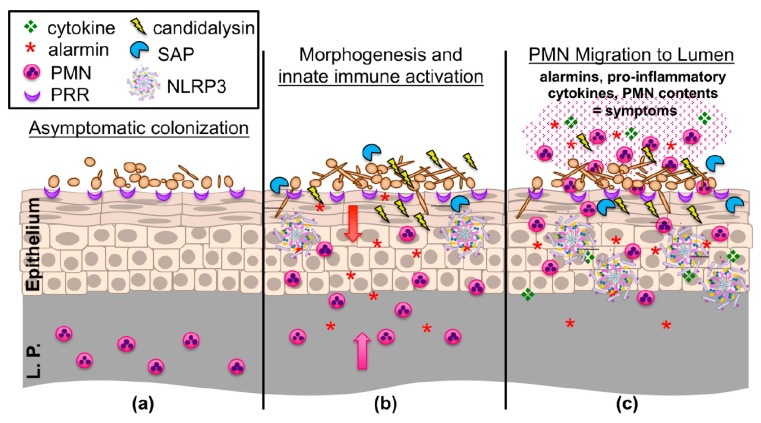
An updated working model of the immunopathogenesis of *C. albicans* vaginitis. (**a**) Yeast forms of *C. albicans* asymptomatically colonize the vaginal epithelium despite the presence of numerous pattern recognition receptors (PRR) on the epithelial surface. (**b**) *C. albicans* begins to undergo the yeast-to-hypha switch under morphogenesis-inducing conditions (e.g., increases in estrogen, elevated vaginal pH, and microbiome disruption). Augmented recognition by PRRs, increased hyphal biomass, expression of hypha-associated virulence factors (candidalysin, secreted aspartyl proteinases (SAPs)) activates NLRP3 inflammasome signaling, eliciting inflammatory cytokines and chemokines (e.g., IL-1β, S100A8/9 alarmins) in the vaginal epithelium, resulting in initial migration of polymorphonuclear leukocytes (PMNs) from the lamina propria (L.P.) to the vaginal lumen. (**c**) Failure to adequately reduce immunopathological triggers results in the continued expression of innate immune effectors by the vaginal epithelium. These initial signals, coupled with the secondary amplification of immune effectors by recruited PMNs, contribute to symptomatic infection and characteristic immunopathology. Figure adapted from: *PLoS Pathog*. 2014 Apr; 10(4): e1003965 [47].

Prior to the initiation of an immune response, *C. albicans* must first be sensed at the mucosal interface. Primarily, this is mediated through pattern recognition receptors (PRR) that exist at the cellular surface of epithelial and innate immune cells. Ligation of these receptors to their cognate ligands (typically *C. albicans* cell wall constituents) induces pro-inflammatory cytokine signaling and the recruitment of both innate and adaptive immune cells to infectious foci via activation of downstream signaling cascades (Figure 1) [45]. Sensing of *C. albicans* is somewhat dependent on morphogenetic switching, as the challenge of vaginal epithelial cell cultures with hypha-defective Δ/Δ*efg1*/Δ/Δ*cph1* mutants results in a significantly reduced release of pro-inflammatory cytokines and a delayed, alternative activation of the mitogen-activated protein kinase (MAPK) system [46].

The role for Toll-like receptor (TLR) and C-type lectin receptor (CLR) families are the most well described with regard to VVC. Despite its clear protective role against invasive infection, discrepant results exist regarding the role for the CLR Dectin-1 that recognizes fungal β-glucan [48]. Attempts to determine the genetic linkage of RVVC with Dectin-1 deficiency (Y238X mutation) in women have yielded disparate results [49,50]. Using Dectin-1-/- mice, one study demonstrated an elevated susceptibility to VVC in C57BL/6 mice, yet a reduced susceptibility in the BALC/c background; these effects were purportedly due to Th17 effectors [51]. Expression of murine Dectin-1 was not found on the vaginal epithelium, suggesting that if Dectin-1 does play a role during VVC, it likely exerts its effects through the hematopoietic lineage [52]. However, a simultaneous blockade of TLR4 and SIGNR1 (Dendritic-Cell-Specific intercellular adhesion molecule-3-Grabbing Non-integrin) reduced the expression of alarmins during fungal challenge in vivo, demonstrating their important role during early inflammatory events [52]. Although strong clinical evidence exists that CARD9-deficiency (an adaptor protein important for broad CLR signaling) confers susceptibility to mucosal fungal infection, including RVVC, mechanistic studies to assess its role in animal models are lacking [53]. Similarly, the loss of all TLR signaling via MyD88 deletion has not been interrogated comprehensively during VVC. Defects in mannose receptor (MR) and mannose-binding lectin (MBL) signaling, that sense the carbohydrate mannan, have also been proposed to mediate susceptibility to fungal infection. A series of clinical studies identified a polymorphism (codon 54) in MBL-2 that associated with RVVC susceptibility [54,55]. Challenge of epithelial cells derived from MR-/- mice resulted in similar levels of inflammatory markers as those from wild-type mice [52]. Thus, more detailed mechanistic studies are required to fully elucidate the role of mannose recognition in the pathogenesis of VVC.

Using a comprehensive systemics genetics approach, a recent study by Jaeger and colleagues identified a polymorphism (rs2919643) in SIGLEC15, a sialic acid-binding lectin present on immune cells and vaginal mucosa, as a susceptibility allele for RVVC [56]. Siglec15 was shown to strongly bind sialic acid on the surface of a variety of *Candida* species and cleavage of sialic acid from the fungal surface using neuraminidase-attenuated immune activation during challenge of blood monocytes. In vivo knockdown of SIGLEC15 transcript resulted in a higher fungal burden and inflammation as compared to control animals, suggesting an important role in controlling infection. However, the polymorphism identified confers a hyper-inflammatory, pathologic phenotype, driving elevated inflammatory markers, including neutrophil recruitment to the vaginal lumen during murine VVC. Thus, attempts to pharmacologically attenuate this pathway to alleviate symptoms in the RVVC population would have to be carefully controlled as to further exacerbate inflammation or prevent fungal clearance.

### 2.3. Invasion and Secreted Virulence Effectors

Aside from signaling through PRRs, the capacity for *C. albicans* to simultaneously elicit tissue damage is another key piece to understanding the full activation of cellular immune responses [57]. *C. albicans* (as well as the NAC species) exert their damage primarily through two major mechanisms: direct invasion by hyphal filaments or secretion of virulence effectors (Figure 1). Hyphae are crucial for breaching mucosal barriers and causing tissue damage. Host membranes can be weakened by degradative enzymes secreted at the hyphal tip and pressure exerted by the elongating filament is then sufficient to penetrate the host cell [41,58]. Wächtler and colleagues demonstrated *C. albicans* attaches to and begins invading vaginal epithelial cells within 3 h and that continual hyphal growth is required for damage [59]. A number of hypha-associated adhesins help anchor *C. albicans* to the mucosal epithelium and are important for mature biofilm formation in the vagina as deletion of the master adhesin regulator *BCR1* impaired biofilm formation in vivo [60]. Aside from binding to host E-cadherin (and other epithelial receptors), Agglutinin-like sequence 3 (Als3p) also acts as an invasin that induces clathrin-dependent endocytosis of *C. albicans* into epithelial cells, further contributing to its invasiveness [61]. The precise role of Als3 during VVC has not yet been established but a majority of vaginal clinical isolates were found to express this gene [62].

Regarding secreted effectors, the secreted aspartyl proteinase (SAP) and lipase gene families are the most studied. The SAPs are a collection of ~10 genes that degrade extracellular proteins to be used as metabolic nitrogen sources [63,64]. Extensive studies have revealed that SAPs demonstrate morphological specificity and pH optima: SAPs 1–3 are largely associated with expression in the yeast cell and at pH 2–5, while SAPs 4-6 are expressed during hyphal growth and are most active at pH 5–7 [65]. Mice intravaginally challenged with a Δ*sap4*5*6* triple mutant or Δ*sap5* single mutant demonstrated moderate but significantly reduced immunopathological markers early during infection [66]. Other studies using rodent models of VVC demonstrate that recombinant Sap2 and Sap6 are capable of driving vaginal inflammation and that a Δ*sap2* mutant is attenuated in pathogenicity [67,68]. However, overexpression of SAP1-6 in hypha-defective Δ/Δ*efg1*/Δ/Δ*cph1* mutants did not elicit mucosal damage or inflammation during murine VVC [69]. Thus, at this time, the role of the SAPs in contributing to VVC immunopathogenesis is somewhat inconclusive and may be dependent on discrepancies in animal model systems, fungal genetic approaches, and protease doses utilized.

While the pathogenic role of SAPs has been extensively studied in *C. albicans*, the lipases have received less attention. Secreted lipases are encoded by a family of 10 genes (*LIP1*-*10*) and serve an important role in the hydrolysis and synthesis of triaclglycerols [70]. Much like the SAPs, they exhibit differential regulation and temporal patterns of expression [71,72]. Heterologous expression of lipases in *Saccharomyces cervevisiae* revealed that *LIP4*, *6*, *8*, and *10* exhibited true lipolytic activity [73]. Deletion of *LIP8* in *C. albicans* or *LIP1* and *LIP2* in *C. parapsilosis* significantly reduced fungal burden and damage biomarkers, while enhancing survival in murine models of systemic infection [74,75]. However, the role of lipases during VVC has not yet been interrogated.

### 2.4. An Indispensable Role for Candidalysin

Recently, the *ECE1* (extent of cell elongation) locus of *C. albicans* has received much attention, as its gene product is crucially important for pathogenicity [76]. Elegant work by the Moyes, Naglik, and Hube labs have demonstrated that the hypha-specific *ECE1* transcript is translated into a 271 amino acid polypeptide, interspersed by seven lysine-arginine (KR) repeats [77]. Modeling and biochemical analyses have revealed that these KR sites are cleavable by the secretory Kex2p protease, resulting in eight peptides of variable length [77,78]. Disruption of these cleavage sites by alanine scanning and use of a Δ/Δ*kex2* mutant confirmed this suspected secretion mechanism [79]. While the role of seven of these peptides remains enigmatic, peptide three (N-SIIGIIMGILGNIPQVIQIIMSIVKAFKGNK) exhibits lytic activity reminiscent of bacterial cytolysins and the capacity to elicit innate inflammatory pathways (e.g., MAPK) in epithelial and endothelial cells. Thus, it was termed ‘candidalysin’ [77].

Disruption of the *ECE1* locus or the region specifically encoding for candidalysin results in severe attenuation of pathogenicity in multiple infection models, including murine invasive and oral candidiasis, zebrafish swim bladder infection, and a variety of cell culture systems [77,80,81,82,83]. Similarly, deletion of *ECE1* or candidalysin significantly reduces immunopathologic markers of infection (neutrophils, pro-inflammatory cytokines, alarmins) and tissue damage during experimental VVC (Figure 1) [81]. Importantly, these deletion mutants displayed no defect in filamentation or altered colonization in vivo, demonstrating that hyphae are insufficient, and that candidalysin is required for driving immunopathogenicity during VVC. Moreover, treatment of A431 vaginal epithelial cells with various concentrations of candidalysin dose-dependently elicited the pro-inflammatory cytokines IL-1α, IL-1β, IL-6, and IL-8, among others, which was largely reflected in the mouse vagina using candidalysin deletion mutants and revertant strains [81]. Therefore, candidalysin has been established as a crucial virulence determinant driving murine VVC using the reference isolate SC5314. However, its role in clinical isolates and during human infection has not yet been resolved. This, along with the absolute requirement of filamentous or pseudofilamentous morphology in driving immunopathology, remain important, unanswered clinical questions [84]. 

### 2.5. A Controversial Role for IL-17 Signaling?

Cleary, candidalysin causes excessive damage at mucosal surfaces, further amplifying innate immune signaling. In vitro and in vivo work suggests that MAPK signaling, engaged via epithelial growth factor receptor (EGFR) phosphorylation, is important for candidalysin-triggered immune activation [85]. Candidalysin also plays a significant role in eliciting IL-17 responses at the oral epithelium, driving a feed-forward inflammatory loop characterized by IL-1α/IL-β responses [86]. These signaling events have not been entirely elucidated yet for VVC. However, an unbiased global transcriptomic approach using RNA-seq to detect differential gene expression in the murine vagina during infection revealed a gene signature that was strongly suggestive of an IL-17 response [66]. Moreover, this gene signature was remarkably similar to that observed during oral candidiasis [87]. Administration of halofuginone, a pharmacologic inhibitor of IL-17 T-helper cell development, resulted in elevated vaginal fungal burdens during murine infection [88]. Despite these strong indicators for a role of IL-17, the use of IL-17RA-/-, IL-22-/-, and IL-23p19-/- (major mediators of the IL-17 axis) knockout mice demonstrated that neither fungal burden nor neutrophil influx differed between those observed in wild-type mice during murine VVC [89]. Because the aforementioned effects of estrogen may impair IL-17 signaling as previously reported, similar experiments using IL-17RA-/-, ACT1-/-, and IL-22-/- mice were conducted in the absence of estrogen. Regardless of estrogen administration, susceptibility to VVC was not altered in knockout compared to wild-type animals, although less consistent colonization was observed in the absence of exogenous estrogen [87]. While the precise role for IL-17 during human infection is not known, congenital or acquired immunodeficiency affecting IL-17 or Th17 cells seemingly does not predispose women to VVC [90,91]. Collectively, these data support an emerging consensus that, despite the strong induction of the Th17 axis, it is dispensable for the immunopathogenesis of VVC.

### 2.6. The NLRP3 Inflammasome: A Key Role in VVC Pathogenesis

A closer look at transcriptional regulator analysis from RNA-seq studies revealed that the NLRP3 inflammasome plays an important role during disease pathogenesis, as IL-1R pathway genes were increased during VVC [66]. The inflammasomes are a collection of cytosolic innate pattern recognition receptors (PRR) that respond via a two-step activation process involving priming of the response via ligation of cell surface PRRs and sensing endogenous and exogenous danger signals [92]. Damage induction ultimately drives inflammasome oligomerization and activation of Caspase-1, leading to cleavage and secretion of IL-1β—the major inflammasome effector. Signaling via IL-1R can overlap with MAPK activation and subsequent pro-inflammatory cytokine production, followed by robust neutrophil recruitment [86,93]. Given these properties and the capacity for candidalysin to induce damage, and a demonstrable role for protection against oral candidiasis, the NLRP3 inflammasome as a partial driver of innate inflammatory events during VVC was plausible [94]. Indeed, independent experiments from different research teams confirmed that neutrophil influx, cytokine, and alarmin secretion were significantly attenuated in NLRP3-/- animals as compared to controls [66,95]. Interestingly, unlike most models of candidiasis where NLRP3 mediates protection, fungal burden in knockouts was not altered during infection, suggesting that, in the case of VVC, NLRP3 drives immunopathogenic inflammation (Figure 1).

A series of studies have further established the NLRP3 inflammasome as an important mediator of innate immune responses induced by *C. albicans*. Prior work established that Δ/Δ*efg1*/Δ/Δ*cph1* mutants, incapable of undergoing the yeast-to-hypha transition, fail to activate IL-1β secretion in macrophage cell lines [96]. More recent work has established that candidalysin is a potent activator of the NLRP3 inflammasome, as treatment of NLRP3-/- or Casp1-/- macrophages with synthetic candidalysin fails to induce IL-1β secretion [80]. Similarly, challenge of wild-type macrophages with Δ/Δ*ece1* or candidalysin deletion mutants abrogated IL-1β processing and secretion [82,97]. These results are in line with those prior, using hypha-defective strains that consequently also do not secrete candidalysin [96]. Work comparing the relative capacity of various *Candida* species to activate NLRP3 revealed that all, aside from *C. albicans*, are relatively poor inducers of these responses likely because they infrequently form hyphae (if at all) and demonstrate significantly reduced *ECE1* expression [31]. This is supported by other work demonstrating that either a high multiplicity of infection or extended infectious time courses are required for the robust inflammasome responses elicited by NAC species [98]. This data could explain why NAC species make up a minority of VVC infections.

Aside from the role of candidalysin in eliciting inflammation via inflammasome signaling, the SAPs, particularly Sap2 and Sap6 have also been observed to activate NLRP3. Work by the Cassone group has shown that treatment of vaginal epithelial cells with recombinant Sap2p is sufficient to activate Caspase-1, induce IL-1β, and drive neutrophil migration, while its blockade with a neutralizing antibody or enzymatic inhibition with pepstatin A is sufficient to reduce these effects [68]. Similar results were also found for Sap6, where vaginal administration of SAPs to mice up-regulated neutrophil chemoattractants and drove sterile inflammation. Moreover, treatment of human monocytes with either rSap2p or rSap6p elicited high levels of NLRP3 inflammasome effectors IL-1β and IL-18, which was strongly attenuated by knockdown of NLRP3 transcripts [99]. While data suggest that rSAPs are capable of eliciting inflammasome signaling during VVC, this has not yet been conclusively demonstrated using NLRP3-/- mice. That said, *C. albicans* possesses multiple, redundant pathogenicity mechanisms for triggering inflammatory processes within the host.

Roles for inflammasome signaling during cell culture infection and VVC are clear and an increasing number of studies highlighting the relevancy of this pathway during human infection are starting to emerge. A large-scale clinical study revealed that women with RVVC exhibited significantly higher IL-1β and lower IL-1RA (IL-1R antagonist) levels in vaginal lavage fluid as compared to healthy controls, suggesting that the NLRP3 inflammasome may be more highly active in the RVVC population and driving exacerbated immunopathology [95]. A much smaller clinical study similarly revealed that NLRP3 and CASP1 expression was higher in vaginal epithelial cells of women with acute symptomatic VVC as compared to asymptomatic or uncolonized control subjects [100]. Interestingly, expression of *ECE1* was significantly higher in the symptomatic as compared to the asymptomatic group, suggesting that candidalysin may be important for eliciting inflammasome signaling and subsequent inflammation. A genetic survey of over 800 patients revealed that increased VVC susceptibility associated with a unique 12/9 genotype that includes distinct polymorphisms in NLRP3 [101]. Inflammatory markers in vaginal fluid demonstrated increased IL-1β and decreased IL-1RA with this genotype under both asymptomatic and symptomatic states, suggesting that host genetic variation in inflammasome (or other pathways) likely influences VVC susceptibility. Higher powered, more comprehensive, genome-wide association studies are desperately needed to help identify genetic markers of vaginitis risk and novel intervention points. 

### 2.7. Estrogen-Dependent Immunomodulation

Despite the robust inflammation and neutrophil recruitment observed during VVC, fungal burdens remain high. This begs the question as to why these cells are recruited to the vaginal lumen if they are, in fact, not protective? The answers may lie in hormonal effects exerted on host immunity. Mice, like humans, progress through a cyclical reproductive cycle characterized by waves of waxing and waning hormones [102]. However, unlike humans, mice do not menstruate but instead recruit neutrophils to the vaginal lumen to debride the endometrium. Under high estrogen conditions, neutrophil migration is arrested in the vaginal stroma, unless *C. albicans* elicit their chemotaxis by re-establishing chemokine gradients [103]. One theory suggests that estrogen creates an immunologically tolerant vaginal environment, to abrogate responsiveness to foreign antigens (e.g., sperm protein) encountered during peak mating receptiveness [104]. Thus, estrogen may promote a transient, lower FRT-specific immunosuppression, allowing for fungal overgrowth.

Fidel and colleagues presented a novel hypothesis referred to as “neutrophil anergy” to help explain neutrophil inactivity in the vagina [105]. C57BL/6 mice are highly susceptible to estrogen-dependent VVC, while CD-1 mice are relatively resistant to vaginal colonization [106,107]. Interestingly, supplementation of cell culture medium with vaginal fluid obtained from C57BL/6 mice significantly impeded neutrophil-mediated killing of *C. albicans* in vitro, while fluid from CD-1 mice was similar to untreated control medium. Moreover, fluid from C57BL/6 mice lost neutrophil inhibition when mice were not administered estrogen. The active compound in C57BL/6 fluid was identified as heparan sulfate, and its effects on PMN activity could be mitigated by degradation with heparanase. Evidently, heparan sulfate is a competitive inhibitor of Mac1, present on neutrophils and interferes with recognition of the fungal surface ligand Pra1p, allowing *C. albicans* to evade killing [107]. Given that heparan sulfate is estrogen-inducible, this could provide a rationale as to why neutrophils are ineffective at clearing *C. albicans* in vivo. Clinical studies are needed to survey heparan sulfate levels in RVVC, symptomatic, and control groups of women in order to further establish this interesting hypothesis.

## 3. A Role for the Microbiome?

A balanced and healthy vaginal microbiome is considered to be important for maintaining female health, further influencing the health of their sexual partners and newborns, as well as the success of pregnancy and delivery [108]. Alteration in vaginal microbiome is a significant risk factor for developing diseases of the FRT, including *Candida* vaginitis [109]. Comprehensive studies on healthy human vaginal microbiome have identified *Lactobacillus spp.* to be the dominant bacterial species by both culture-dependent and -independent methods [110,111,112,113]. Although absolute numbers vary, due to different endogenous and exogenous conditions (age, lifestyle, pregnancy, hormone levels, etc.), the relative abundance of *Lactobacillus spp.* in the human vagina is >70% [114]. Among the five groups of human vaginal communities from reproductive-age women, I, II, III, and V were dominated by *Lactobacillus crispatus*, *L. gasseri*, *L. iners*, and *L. jensenii*, respectively [113]. Through the excretion of metabolic byproducts (as discussed below) and subsequent acidification of the vaginal microenvironment, *Lactobacillus spp.* are considered to help maintain homeostasis by preventing the colonization or overgrowth of pathogens. 

Studies attempting to define the contribution of the microbiome to *Candida* vaginitis have resulted in disparate conclusions, mainly regarding the role of *Lactobacillus spp.* in preventing pathogen (*Candida* spp.) colonization. Interestingly, the vaginal microbiomes of women suffering from VVC, *Chlamydia trachomatis*, or bacterial vaginosis exhibit distinct profiles that further differ from those of otherwise healthy women [109]. Several studies have suggested vaginal dysbiosis during VVC, with reduced *Lactobacillus* colonization rates or patterns [109,115,116,117]. Although *Lactobacillus* remained dominant at the genus level, reduced rates (<67.5%) and altered species (*L. crispatus* progressively replaced by *L. iners*) were found during disease states [109,113]. The vaginal microbiome of VVC patients following antifungal treatment were also distinct, demonstrating similarities to both healthy and dysbiotic microbiomes, which might represent a transition between these states [116]. However, the protective role of *Lactobacillus spp.* against *Candida* spp. has been questioned, as high densities of lactobacilli often accompany *Candida* in the vagina during active vaginitis [117,118]. In fact, even elevated levels of *Lactobacillus spp.* (*L. iners* and *L. crispatus*) can be observed during VVC, broadly suggesting the failure of *Lactobacillus spp.* to suppress *Candida* colonization [118]. Other clinical studies have failed to provide evidence of an altered or abnormal vaginal microbiota in women suffering from *Candida* vaginitis, despite the maintenance of *Lactobacillus* abundance [119,120]. Discrepancies among studies may be due to inherent variables (e.g., patient geography, age, symptoms), as well as approaches used for sample collection, storage, culturing and identification. 

Despite the unclear effect of *Lacotbacillus* in vivo, a majority of in vitro and animal studies have revealed that *Lactobacillus spp.* exerts an inhibitory effect on the growth, morphological transition, virulence, and biofilm formation of *C. albicans* [121,122,123]. The metabolites of *Lactobacillus spp.* including organic acids, hydrogen peroxide, bacteriocins, and biosurfactants, all contribute to these antifungal effects [124,125]. However, only specific *Lactobacillus* strains are capable of producing these effectors in quantities required for antifungal activity, which possibly explains the failure of some vaginal lactobacilli to suppress *Candida* colonization. The transition of *C. albicans* from yeast to hypha is inhibited at low pH (< 4.5) [126]. Thus, the secretion of lactic and other organic acids is believed to be the main mechanism by which lactobacilli impede candidal virulence. In support of this, down-regulation of hypha-specific transcriptional regulatory genes (*EFG1*, *BCR1*, and *CPH1*, etc.) and virulence related genes (*HWP1*, *ALS3*, *ECE1*, and *SAP5*) were observed during co-culture with lactobacilli or treatment with spent culture supernatant [125,127]. Lactobacilli were also reported to compete with the attachment of *C. albicans* to host cells, resulting in reduced adhesion to epithelial surfaces [128]. *Lactobacillus* strains originating from sites other than the vagina have also been frequently observed to exhibit strong anti-*Candida* properties, suggesting more global growth-inhibitory effects [129,130]. While the clinical data regarding *Lactobacillus* colonization and its role in preventing VVC are unclear, some clinical studies using lactobacilli as probiotic colonizers have shown promising protective effects [131,132]. 

## 4. Established and Novel Treatment Modalities

There are numerous current therapeutic options available for the treatment of VVC that vary based on uncomplicated or complicated (e.g., RVVC, NAC species) presentation. Treatments mainly consist of prescription oral dosage forms, or over-the-counter topical preparations, or vaginal suppositories [133]. Azoles are the most common class of antifungal drugs used to treat vaginal candidiasis due to their good bioavailability, antifungal efficacy, and relative safety. However, the azole class exhibits static activity, thus requiring efficient host immune function to aid in the resolution of symptoms. Common over-the-counter azoles and treatments are: clotrimazole 1%–4% topical cream for daily usage between 1–7 days, miconazole 2%–4% cream for 3–7 days or 100–1000 mg suppository for 1–7 days, and tioconazole 6.5% intravaginal ointment in a single application [134]. Prescription azole agents include butoconazole 2% topical cream applied intravaginally, terconazole 0.4%–0.8% daily for 7 days or 80 mg vaginal suppository (one every day for 3 days) and fluconazole 150 mg single oral dose [134]. Patients with NAC-related or recurrent VVC are often prescribed a longer duration of initial topical (e.g., 7–14 days) or oral (100–200 mg every 72 h for three doses) therapy [133]. Women suffering from severe or highly recurrent disease may take azole drugs weekly to prevent symptomatic episodes [135]. While oral and systemic isolates of *C. albicans* can exhibit high levels of azole resistance, treatment of VVC with this antifungal class is largely effective and overall resistance rates remain low [136]. In the event that resistance is detected, less conventional therapies including boric acid suppositories and creams containing nystatin, amphotericin B, or flucytosine, are indicated [17].

Topical products containing local anesthetics (5% benzocaine) and antiseptics (2% resorcinol) to relieve itching and burning also exist, but many women complain that they actually induce stinging and burning during application [137]. Thus, new treatment modalities are desperately needed to resolve disease symptoms in a timelier fashion. Given that the NLRP3 inflammasome appears to play a major role in driving murine and clinical immunopathology, strategies to attenuate the activation of this molecular complex are appealing. Administration of the IL-1R antagonist anakinra, to interfere with signaling of the inflammasome effector IL-1β, significantly reduced neutrophil recruitment and pro-inflammatory cytokines in a mouse model of VVC [95]. Administration of the anti-diabetic sulfonylurea glyburide (and established NLPR3 inhibitor) to mice attenuated neutrophil recruitment during VVC [66]. Moreover, treatment of macrophage-like cells with various second-generation sulfonylurea drugs and the highly potent NLRP3 antagonist MCC-950 (glyburide, glisoxepide, gliquidone, and glimepiride) dose-dependently inhibited NLRP3-dependent IL-1β secretion, suggesting that these anti-diabetics could be repurposed as potential anti-inflammatory agents [97]. Collectively, these data suggest that targeting the NLRP3 inflammasome during VVC may be a rational therapeutic option for disease management.

Probiotic therapy, or the administration of beneficial microbes to the mucosal surface, has been suggested for the treatment or prevention of *Candida* vaginitis. A study by Pericolini and colleagues has found that introduction of *S. cerevisiae* to the murine vagina could help protect against *C. albicans* challenge [138]. Purportedly, anti-candidal effects included adherence inhibition, accelerated clearance, blockade of the yeast-to-hypha transition, and reduced expression of established virulence effectors (e.g., SAPs). Unsurprisingly, this was correlated with reduced immunopathological markers of infection. Vaginal epithelial cells were also found to exhibit less exfoliation and reduced damage biomarkers when *S. cerevisiae* was introduced intravaginally. Whether these effects are strain-specific or effective against a broad array of *Candida* species or isolates remains unknown. Given that *S. cerevisiae* has been shown to cause vaginitis-like symptoms in a limited number of women, significant clinical testing would be required to rule out any potential negative consequence of abundant vaginal yeast colonization [139].

Lastly, the holy grail of reducing the incidence of VVC lies in the development and deployment of a safe, efficacious, and long-lasting vaccine. A significant amount of work by Edwards and colleagues has made remarkable strides in this arena with the advent of the NDV-3 vaccine [140]. NDV-3 is composed of a recombinant epitope of Als3p delivered in alum adjuvant. It has demonstrated excellent efficacy in dose-dependently reducing fungal burdens several logs in the mouse model of VVC [141]. NDV-3 exerts its protective effects by priming both humoral and adaptive immune responses, as protection was lost in both B- and T-cell-deficient mice. Importantly, reduced fungal burdens correlated with decreased neutrophils levels, indicating that NDV-3 administration should also temper symptomatic episodes. Administration in human volunteers established that the vaccine was well-tolerated and highly immunogenic, generating long-lasting memory B- (IgG, IgA1) and T-cell (IFNy, IL-17A) responses that were enhanced with a booster dose [140]. Recent data from a Phase 1b/2a clinical trial offered significant protection against RVVC in women < 40 years of age [142]. Serum recovered from immune subjects that remained VVC-free, as compared to those experiencing relapse, exhibited high Als3-specific IgG titers, which, when pre-absorbed to *C. albicans,* inhibited its filamentation, epithelial cell invasiveness, and biofilm formation. Aside from NDV-3, a truncated rSAP2 protein incorporated into influenza virosomes, termed PEV7, was also shown to confer protection against VVC in rats, presumably via antibody-mediated effects [143]. While these collective data are promising, important studies to delineate boosting strategies, include other antigenic determinants, or alter adjuvant choice, will help optimize the efficacy of a globally protective vaginitis vaccine.

## 5. Conclusions and Burning Questions

*Candida* vaginitis is a universally important disease with wide-reaching effects on the overall physical and mental health of women, coupled with significant impacts on the medical and work-force economy. Recent advances in our understanding of VVC as an immunopathology and the mechanistic roles of candidalysin, inflammasome, and PRR-mediated signaling at the vaginal mucosa have opened up multiple avenues for scientific exploration. While progress has been impressive, several important questions and challenges remain.

Our understanding of the host and fungal molecular mechanisms contributing to VVC pathogenesis have largely been identified using the *C. albicans* reference isolate SC5314. However, work with various clinical isolates, especially those obtained from women who are asymptomatically colonized or from vaginitis cases, should be a high priority to determine the relevancy of signaling pathways and effector mechanisms. For example, SC5314 expresses very high levels of *ECE1*, but is this representative of the majority of clinical isolates? Relatedly, how much candidalysin does *C. albicans* actually secrete, and are the relatively large concentrations used in cell culture assays physiologically representative? As the propensity to undergo morphogenetic switching across clinical isolates varies considerably, surely this, too, will impact pathogenicity [144]. Delineating genetic mechanisms that underlie vaginal strain-to-strain virulence, through the use of large-scale whole genome sequencing and genome-wide association studies, may identify pivotal regulons and novel therapeutic fungal targets to reduce VVC incidence.

Heterogenous virulence among *Candida* isolates likely plays a role in distinguishing asymptomatic colonization from symptomatic infection. However, there are equally likely host genetic mechanisms at play that intertwine with hormonal regulation, lifestyle, pharmacologic influence, and microbiome composition. Appropriately controlling for all such factors, even in well-controlled clinical studies, is incredibly difficult. Moreover, early immunologic or pathogenic events may be overlooked in such studies, as the symptomatic episode commands focus. Race may also play a factor in disease severity, as African American women report symptoms more frequently and with higher incidence than Caucasian women [145,146]. These disparities further complicate accurate modeling of the natural history of VVC pathogenesis. Although it is difficult to recruit volunteers for a sufficiently powered study, more live-challenge trials with controlled inocula and isogenic strains are needed to comprehensively elucidate the mechanisms leading to human VVC.

While the animal model of VVC has been an indispensable tool for dissecting mechanisms contributing to VVC, it does not come without caveats [147]. Firstly, the vaginal pH of women is generally acidic (<4.5), while the pH of mice is more neutral (~7.0). Mice, unlike women, do not naturally harbor *C. albicans* in the vagina, so introduction of fungal inocula represents a first-time encounter that may result in exacerbated immune reactions. Moreover, the delivery of a bolus dose of fungus is artificial and does not represent the outgrowth of endogenous *Candida* flora occurring in women. Mice also do not exhibit clinical signs of VVC (scratching, itching, vaginal redness). Thus, it is imperative to establish whether these responses are merely correlates of infection or actually drive disease symptoms. Caveats aside, the excellent availability of genetic knockouts, immunological reagents, and low cost will keep the mouse as the standard model system for studying VVC. However, improvements can be made. Identifying a mouse line(s) that more accurately presents with disease symptoms would be immensely useful. Perhaps the use of humanized mice produced to maintain human immune cells would provide novel insights into disease pathogenesis. The CRISPR/Cas9 revolution should also make engineering genetic variants, especially those identified with high frequency in RVVC subjects, more amenable to reflect functional differences in human alleles. Lastly, a model of recurrent disease has been a long-time need; unfortunately, until the factors that contribute to RVVC are better understood, this will likely be unattainable.

**Figure 2:**
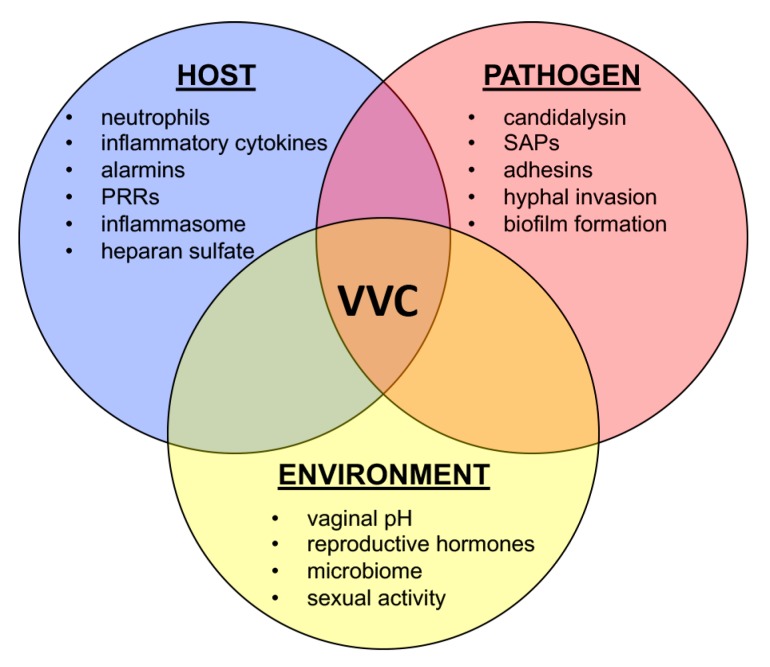
Vulvovaginal candidiasis (VVC) is a multifactorial disease. Multiple inputs from host (blue), pathogen (red), and environment (yellow) are required to drive disease onset and symptomatic infection. Each circle represents major contributing factors to the immunopathogenesis of VVC.

*Candida* vaginitis is a complicated disease, whose symptoms are governed by the intersection of host physiology, fungal biology, and the immunological response (Figure 2). The recognition of VVC as an immunopathology was a paradigm-shift that cannot be overstated [40]. While we have gained significant understanding of both host and fungal factors driving pathogenesis through this lens, there is still much progress to be made. As the global incidence of VVC is predicted to rise by tens of millions in the following decade, novel treatment modalities, improved diagnostics, and broad-scale pathogenomics studies will be required to lessen the impact of this currently unmet clinical need [3].

## Figures and Tables

**Table 1:** Estimated annual global incidence of candidiasis.

Candidiasis Route	Avg. Incidence	Approx. Target Population ^1^	Approx. Individuals Impacted	Refs.
**Invasive**	0.009%	7,800,000,000	**702,000**	[8]
**Oropharyngeal**	-	-	**15,337,200**	
HIV/AIDS ^2^	72.5%	15,200,000	11,020,000	[9,10]
Head/neck cancer	40%	650,000	260,000	[11,12]
Is Organ transplant	11.6%	100,800	11,690	[13]
Infants (< 6 mos.)	6%	67,425,000 ^3^	4,045,500	[14,15,16]
**Recurrent vulvovaginal**	8%	1,682,200,000	**134,560,000**	[17]

^1^ Based on references cited. ^2^ Based on global population living with HIV/AIDS and not currently on retroviral therapy. ^3^ Calculated as: (crude global birth rate - global infant mortality rate)/2.
